# On the Road to Circular Polymer Brushes: Challenges and Prospects

**DOI:** 10.1021/acs.langmuir.3c03683

**Published:** 2024-04-01

**Authors:** Maria Brió Pérez, Frederik R. Wurm, Sissi de Beer

**Affiliations:** Department of Molecules & Materials, MESA+ Institute, University of Twente, P.O. Box 217, 7500 AE Enschede, The Netherlands

## Abstract

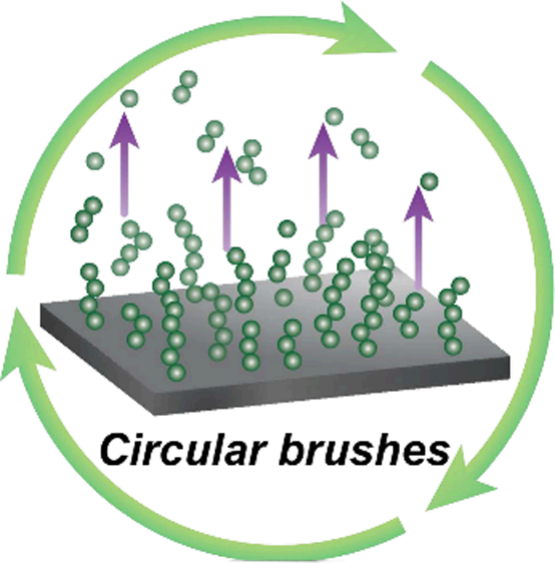

Polymer brushes are unique surface coatings that have been of high interest in research for the past decades due to their covalent tethering to surfaces and the broad spectrum of polymers that can be grafted to or grafted from various surfaces. Modification of surfaces with brushes may provide lubricious and/or antifouling properties, and they can also potentially be used in many application fields due to their high responsiveness toward certain stimuli. Generally, polymer brushes are long-lasting coatings, while their end-of-life has to date largely been neglected. Therefore, it is important to consider additional design methodologies to produce circular brushes, which will degrade after a certain period of time such that surfaces can be reused, and the potentially obtained monomers may be used again to synthesize new brushes. In this Perspective, we aim to tackle and understand the challenges to translate the knowledge on degradation and chemical recycling of bulk polymers toward circular polymer brushes. We summarized the recent developments on (bio)degradable polymer brushes and the challenges that are to be tackled toward their potential implementation as circular coatings.

## Introduction

Polymer brushes are formed when polymers are tethered to a surface at densities that are high enough for them to stretch away from the substrate. These brushes are popular coatings because they allow for advanced control of interfacial properties, such as wetting, adhesion, and friction, both in liquid^1^ and in air.^2^ This is relevant for a broad variety of applications, ranging from sensing^3^ to preventing surface fouling.^4^

There are different methods to prepare brushes, by either “grafting to” or “grafting from” techniques. Currently, surface-initiated controlled radical polymerization (SI-CRP) techniques^5^ are utilized most commonly because they produce well-defined and high-density polymer brushes. Examples of these techniques are atom transfer radical polymerization (ATRP), nitroxide-mediated polymerization (NMP), or reversible addition–fragmentation chain transfer (RAFT). An additional advantage of these techniques is that the synthesized polymers typically have very stable C–C bonds, which makes the brush coatings stable against degradation. However, this stability poses challenges in adapting to a circular economy for both surfaces and polymer coatings. Especially, the accumulation of polymer waste and the release of microplastics from polymer materials are current societal concerns. These issues lead to environmental pollution, health risks, and challenges in waste management and ecological balance.^6^ To mitigate this, it has become more and more relevant to design polymeric materials and coatings that can degrade or be removed by depolymerization (i.e., chemical recycling).

Although there are some reports on degradable brushes for, e.g., biomedical applications or circularity arguments, these articles do not address the actual degradation process. Research on degradable bulk polymers is more widespread than for degradable polymer brushes due to a longer history of use and research and the pressing environmental concerns related to plastic waste. Commonly used recycling approaches for bulk polymer coatings focus on the reusability of the surfaces, whereas the coated polymers are disposed such that a new coating may be added to the same substrate.^7^ This approach is adopted because within commodity plastics, only very few polyvinyls such as poly(vinyl alcohol) (PVA) can undergo partial degradation under certain conditions.^8^ Other aromatic polymers like poly(styrene) may undergo UV-initiated degradation, followed by partial backbone cleavage.^9^ Thus, designing polymers that can break down completely under specific environmental conditions is essential such that both surfaces and coatings become circular. For the development of degradable or recyclable coatings, alternative polymer types, such as polyesters, have been proposed and are currently used as biodegradable polymers. These include polymers such as poly(lactic acid) (PLA), being the most prevalent one (24% of the global production capacity of biodegradable polymers), polyhydroxyalkonates (PHAs), and polycaprolactone (PCL), among others.^10^

Degradable polymer brushes have demonstrated a great potential across diverse fields, ranging from medicine to high-end technology as well as addressing sustainability issues. These materials have been proposed in drug delivery and tissue engineering, as surgical sutures, degradable scaffolds, and bioactive coatings.^11−13^ Their applicability extends further to microelectronic devices, as nanostructures for separation technologies, catalysis, and sensing.^14−16^ They have also been used in marine antifouling coatings, inducing the self-renewal of surfaces by brush degradation.^17^

The development of biodegradable polymer brushes represents a significant step toward sustainable material science, addressing the urgent need for responsive thin films that will not contribute to the growing problem of plastic waste and microplastic pollution. By adjusting the chemical composition of these brushes, researchers aim to develop materials that combine desirable surface properties and functionality that can degrade safely and effectively during their use. Despite the advances on degradable brush synthesis, there has been a lack of systematic studies or characterization on the degradation processes of these coatings, even though they can be expected to deviate from bulk degradation.^18^ This area of research on polymer brushes is expected to grow by further addressing fundamental challenges and exploring the applicability of these coatings.

In this Perspective, we will provide a brief overview of the recent advances within the field of (bio)degradable homopolymer brushes and the assessment of their degradation, with a particular emphasis on the challenges toward their future implementation. In recent years, significant progress has been made within this field, including the development of thicker and easier-to-characterize degradable brushes, the evaluation and fundamental understanding of their degradation mechanisms, and evaluation of their feasible use in various applications.^18−20^ Together with the established synthetic protocols and knowledge for bulk materials, we believe a transition toward circular polymer brush coatings is achievable. Although understanding the depolymerization of brushes is key for the development of truly circular brush coatings, this topic has received relatively limited attention in the existing body of literature. Hence, the feasibility of applying bulk depolymerization techniques to brush coatings will be discussed in the upcoming “Moving Forward” section.

## Degradation

Polymer degradation is influenced not only by the chemical structure of each polymer but also by environmental factors, which have been thoroughly reviewed in the literature.^21,22^ The susceptibility of a polymer to hydrolysis largely depends on its chemical composition; polymers containing hydrolytically labile linkages such as ester, amide, and carbonate bonds are more prone to degradation than those with more stable bonds like ethers or C–C bonds. Temperature and pH further affect the degradation processes of polymers; at higher temperatures hydrolysis reactions are accelerated.^23,24^

Additionally, the acidity or basicity of the degradation media may lower the activation energy for bond breaking and thereby facilitate the breakdown of susceptible bonds, which is particularly relevant in settings where the material encounters fluctuating pH levels.^18,19,23^

**Figure 1:**
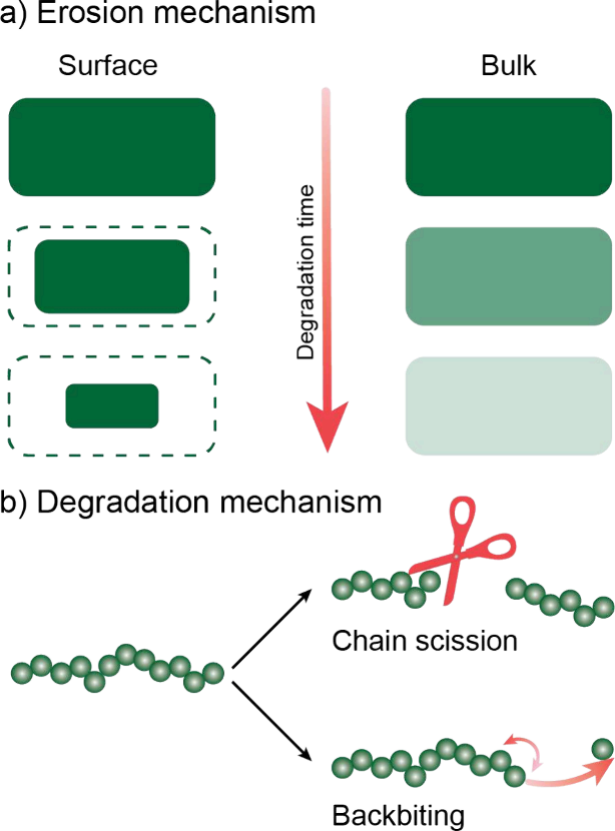
Illustrative representation of (a) erosion types and (b) degradation mechanisms undergone by degradable polymers.

Macroscopically, polymer hydrolysis may proceed via surface or bulk erosion (Figure 1a). The hydrophobicity and crystallinity of the polymer, together with the rate of diffusion of water into the polymer, will determine the erosion mechanism type. Furthermore, certain polymers may shift their erosion mechanism when their thickness decreases below a certain critical thickness.^21^ On a molecular level, backbiting or random chain scission degradation mechanisms have been observed per polymer type. In the case of PLA, the backbiting mechanism is predominant under basic conditions, whereas under acidic conditions it degrades via random chain scission. Other polymers such as PCL degrade mainly via random chain scission in both basic and acidic conditions (Figure 1b).^22^ These complex mechanisms highlight that environmental conditions dictate degradation kinetics. Thus, it is crucial to have an in-depth understanding on the degradation processes undergone by these polymers for each given application.

## Depolymerization

Recent research in depolymerization has uncovered strategies on the chemical recycling of plastics into their constituent monomers, which can be harvested for repolymerization.

Polymers produced from recovered monomer feedstocks should sustain their properties, thus recovering the value of the initial material while mitigating environmental effects. Depolymerization can be achieved through various mechanisms, including thermal, chemical, and enzymatic processes, which have been reviewed elsewhere.^25^ During depolymerization, a physical or chemical trigger would either expose the chain ends of the polymers or induce chain scission in polymer backbones to generate new chain ends which are capable of unzipping.^26^ Depolymerizable polymers may be used in controlled-release systems, where a controlled breakdown of the polymer would gradually release the encapsulated substances.^25,26^

The evaluation of brush depolymerization has remained relatively unexplored due to the harsh conditions that are often utilized in conventional chemical recycling approaches, which can also potentially damage the surfaces where these brushes are coated. To date, within these topics, research on grafted polymer brushes has primarily focused on qualitative assessments of their degradation. This is due to synthetic difficulties together with the reduced thickness of brush coatings (nanometer to submicrometer scale), which complicates their characterization and the assessment of their gradual degradation. Moreover, the susceptibility of brushes to degrafting reactions^27^ may result in direct micro-/nanoplastic contamination derived from the cleaved polymer brush chains. This is particularly relevant for brush-coated nanoparticles or porous materials, e.g., in potential sensing or separation technologies, where an increased volume of polymer chains per surface area would be released to the environment.

## (Bio)degradable Brushes

**Figure 2:**
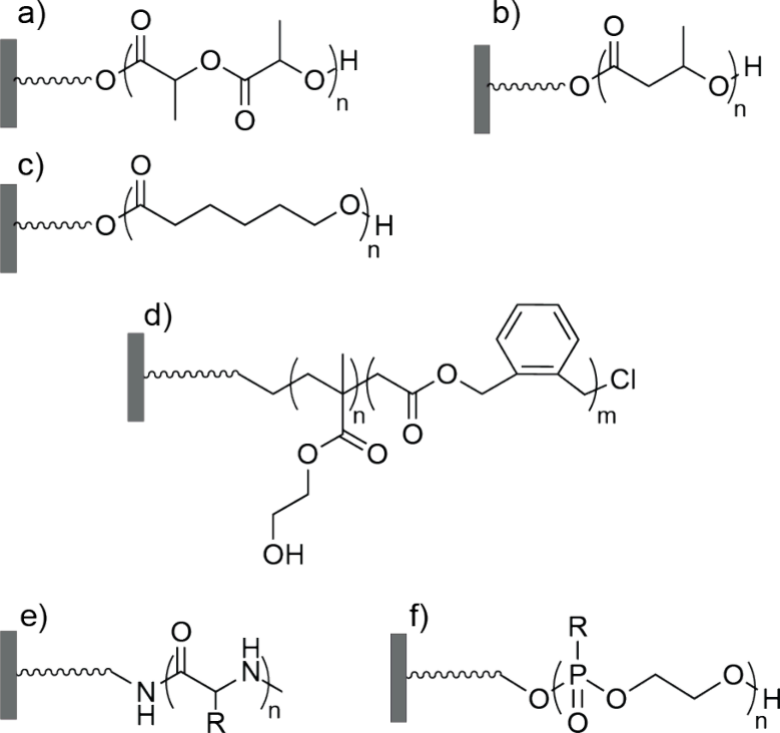
Biodegradable polymer brush types shown in the literature: (a) poly(lactic acid) (PLA), (b) polyhydroxybutyrate (PHB), (c) polycaprolactone (PCL), (d) poly(PEGMA_*n*_-*co*-BMDO_*m*_), (e) polypeptides, and (f) polyphosphoesters, with R indicating adjustable side-chain groups.

The currently available research on degradable brushes is based on polyesters^18,19,23,24,28,29^ and on polypeptides.^20^ Polyester brushes are most prevalent in the literature and are commonly synthesized from cyclic ester monomers with varied hydrolytic stability, such as lactide, butyrolactone, or ϵ-caprolactone, which can be polymerized via surface-initiated anionic ring-opening polymerization (SI-AROP) (Figure 2a–c).

These have commonly led to PLA and PCL polymer brush coatings with ultralow thicknesses (ca. 10 nm) after long polymerization times.^23,24,29^ A reduced thickness brings difficulties in the characterization of the coating throughout the degradation process due to a compromised sensitivity on thickness measurements when evaluating changes of a fraction of a nanometer. In addition, there are no literature works to our knowledge that have analyzed brush degradation products due to the highly reduced volumes that may be extracted during brush degradation processes.

**Figure 3:**
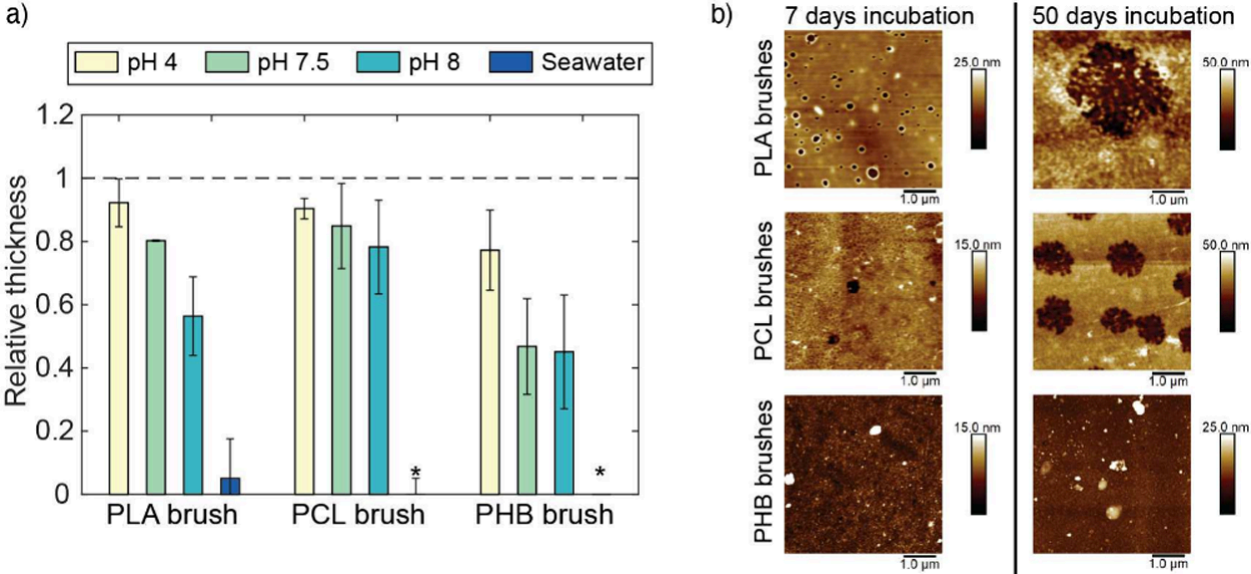
(a) Degradation of polyester brushes, shown by changes in relative thickness for each brush after 50 days of incubation in buffered aqueous solutions of varying pH and seawater, with “∗” indicating total degradation and the dashed line showing the initial relative thickness. (b) AFM morphology images of PLA (top), PCL (middle), and PHB (bottom) polymer brushes, after 7 (left) and 50 (right) incubation days in a PBS solution of pH 7.5. Adapted with permission from ref (18).

Recently, we showed that by selecting the appropriate macroinitiators, it is also possible to graft polyester brushes of varying hydrolytical stability and moderate thicknesses (up to 50 nm) from polyol-based stable macroinitiators on silicon surfaces with high control and reproducibility.^18^ These brushes showed an enhanced durability (months) in comparison to the ultrathin counterparts (days),^23,24^ while still being fully degradable in artificial seawater in less than 15 days (Figure 3a).

The synthesis of degradable copolymer polyester-based brushes has also been successfully shown either by using two different lactones^28^ or by combining conventional monomers used in SI-CRP such as poly(ethylene glycol) methacrylate (PEGMA) with cyclic ketene acetal monomers such as 5,6-benzo-2-methylene-1,3-dioxepane (BMDO), which provide hydrolyzable ester bonds (Figure 2d).^19^ These copolymer brush types showed shorter degradation times with decreasing pH and increasing BMDO content. However, only the breaking points provided by the BMDO groups will hydrolyze, with the PEGMA oligomers being the remaining degradation products, which do not undergo further degradation. Overall, polyester (homo)- and (co)polymer brushes with adjustable hydrolytical stability may pose as interesting candidates in the development of degradable coatings for controlled drug delivery or tissue engineering.

Polypeptide brushes, which are composed of amino acid-based polymers grafted onto surfaces, are a relatively recent development in the field of biomimetic polymers. The controlled ring-opening polymerization of α-amino acid *N*-carboxyanhydrides (NCA-ROP) has led to the synthesis of polypeptide brushes with tunable thickness between 4 and 40 nm (Figure 2e).^20^ Due to the large family of amino acids, synthetic polypeptides can be tailored to display a diverse range of physicochemical properties, while maintaining their inherent biocompatibility and (bio)degradability. In addition, the secondary structure of polypeptide brushes presents an opportunity to develop new functional materials with hierarchical structures.

Despite their novelty, the field of surface-grafting to form polypeptide films is rapidly expanding toward their applicability in various fields, from tissue engineering to biosensors and catalysis.^20^ However, there is a need for further fundamental understanding of these materials, which remains unexplored. These include addressing challenges such as the lack of control over the primary amino acid sequence, side reactions, the formation of nongrafted films, and the evaluation of their metabolism, degradation, and potential toxicity as brush coatings.

Very recently, polyphosphonate brushes have been proposed as a new biodegradable platform.^30^ These polymers have a unique backbone containing pentavalent phosphorus atoms, which enable the design of modular structures, together with the inherent degradability provided by the ester bonds (Figure 2f). This facilitates chemical modifications around the central phosphorus, allowing the production of polymers with highly varied properties and chemical functionalities, such as hydrophilicity and thermoresponsivity.^31^ This way, controlled degradation mechanisms with adjustable degradation times can be achieved with these polymers in the bulk and also as polymer brush coatings.

Although labeled as degradable brush coatings, the degradation process underwent by brushes is commonly overlooked, and it is assumed to be comparable to the same polymers in bulk.^13,20^ This does not always hold true because given the reduced thicknesses of brush coatings, chain confinement, and structural variations, their erosion and degradation mechanisms may vary from the ones observed for the same polymers in bulk.^18,23^ In order to translate the available knowledge on degradable bulk polymers to degradable brushes, there are multiple challenges to be tackled. These encompass molecular weight and grafting density considerations, the susceptibility of brushes to degrafting reactions, the reusability of surfaces for repeated brush growth, and the chemical recycling of the brush coatings.

## What Are the Challenges?

**Figure 4:**
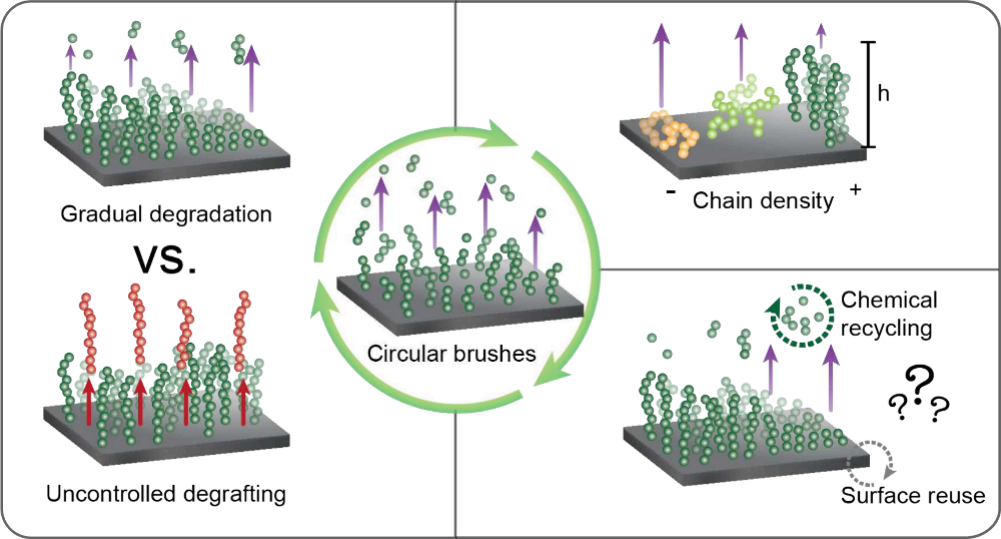
Overview of the challenges and strategies for the development of degradable brushes.

When comparing bulk polymers to polymer brushes, it is important to consider their structural differences and additional challenges (Figure 4). The molecular weight of the polymer chains and their density in the brush layer will affect not only the surface properties and functionality but also their degradability and degradation mechanism. Unique to polymer brushes are degrafting reactions, which are crucial to consider. Additionally, their potential for surface reusability is a key factor in the design of degradable brushes. These aspects are vital for optimal brush design and will be explored in the next section.

### Molecular Weight and Grafting Density Determination

The molecular weight of brushes grown from surfaces is often considered to be analogous to the one of the free polymers grown from sacrificial initiators in the same reaction media, which does not always provide accurate results.^5^

Alternatively, the molecular weight of grafted polymer brushes can be obtained by cleaving the polymers from the surface followed by gel permeation chromatography (GPC) or size exclusion chromatography (SEC) analysis.^32^ Cleaving reactions use strong acids which may lead to unwanted bond breaking and also result in very small amounts of polymer to analyze, challenging the characterization of the molecular weight of these coatings. Only in the case of brush-coated nanoparticles or porous materials, the increased surface area compared to flat surfaces allows for a higher density of cleaved brushes for molecular weight determination.^16^

In response to these challenges, nondestructive techniques for molecular weight determination of brushes have been proposed. These involve indirect molecular weight determinations via atomic force microscopy (AFM)^33^ or single-molecule force spectroscopy (SMFS).^34^ AFM is commonly used to image the surface morphology and density of brushes, and it has also been used to estimate molecular weight and polydispersity. By stretching individual chains away from the grafting surface with an AFM tip and estimating the contour length of the chain from the separation at which the bond ruptures over the grafting surface, we have been able to obtain molecular weight and dispersity values using the length and molar mass of the monomer.

Less common direct molecular determinations with mass spectrometric techniques also have promise for polymer characterization. Time-of-flight secondary ion mass spectrometry (ToF-SIMS) techniques have shown particularly useful and straightforward analysis on the molecular weight and its distribution of polymer brush fragments.^35^

Molecular weight determination of polymer brushes has critical implications for calculating the grafting density, which is a parameter that must be defined in order to unambiguously determine whether surface-tethered polymers exist within the brush regime. The conformations of brushes are dictated by a complex interaction of factors, including the molecular weight and grafting density, solvent quality and type, curvature, and morphology of the substrate. Thus, far, the assessment of grafting densities has proven experimentally challenging.^32^

The concentration of initiators that are present on a coated surface in grafting-from strategies is significantly lower than the one used for bulk polymerizations, but these initiators are typically densely packed in small surface areas. Monte Carlo simulations have shown that polymer brushes have lower molecular weight and higher dispersity than bulk polymers, especially for systems with high grafting densities, due to a higher chain termination probability.^36^ This is in agreement with simple kinetic models.^37^ However, multiple experimental observations show the opposite result, meaning that not all effects are captured by these simulations.^5^

It is well-known that both molecular weight and grafting density have a strong effect on the swelling of polymer brushes.^38^ The grafting density affects the swelling ratio, and the chain length determines the amount of solvent that will be absorbed. Thus, we expect that these two parameters will also strongly influence the degradation behavior of the polymer brushes. Therefore, accurate measurements of molecular weight and grafting density are crucial, particularly in applications in which degradation characteristics are of significant use.

### Degradation vs Degrafting

An additional challenge in the characterization of polymer brush degradation is that brushes are known to break at their surface bonds when these are not sufficiently stable,^38^ already under mild conditions such as humid air.^39^ The most common labile surface bonds on model surfaces are based on silanes or thiols that are previously bound to a substrate.^27^ If the brushes suffer from degrafting, then one cannot attribute a decrease in brush height to polymer degradation. Application-wise degrafting reactions would be detrimental for preserving the functionality provided by the brushes. In contrast, surfaces with controlled degradation and no chain cleavage would regularly release a fresh interface of the same polymer coating. We note that there might be property changes due to a potentially increased dispersity^40^ that might occur during degrafting. Yet, these changes are typically smaller than the effects of the decrease in grafting density that occurs during degrafting. Thus, for degradable polymer brushes, synthetic strategies for stable polymer brushes that limit degrafting should be utilized. These strategies remain synthetically challenging. Yet, they are relevant for a reliable long-term application of polymer brushes. Some of the proposed surface anchors for stable polymer brush grafting which have been reviewed are based on polydopamine (PDA), polyphenols, or poly(glycidyl methacrylate) (PGMA), which strongly bind to a wide variety of surfaces.^27^

Following these strategies, it has been possible to observe distinct erosion mechanisms on polyester brushes at varying pH. Here, incubation in pH 7.5 buffered solutions led to morphology changes in PLA and PCL brushes due to a transition in the erosion mechanism from bulk to surface erosion, evidenced by the formation and growth of voids across the coating (Figure 3b). This transition was not observed in PHB brushes, which only exhibited significant surface roughness changes over time, indicative of a surface erosion mechanism. Together with the degradation kinetic profiles, backbiting was the assigned degradation mechanism for PLA brushes and chain scission for PCL and PHB brushes.^18^ This indicates that degradation and erosion mechanisms may vary per brush type and slight environmental changes. Therefore, it is necessary to further study in detail how degradation processes occur in brushes in order to develop a wider variety of biodegradable brush coatings.

### Surface Reusability

Reusing surfaces after brush degradation is a multifaceted approach that promotes sustainability and a more efficient use of resources. This practice would significantly reduce the environmental impact by minimizing the need for new surfaces required for brush growth and their accumulation. However, it is commonly overlooked due to the limitations that it encompasses.

The most straightforward method involves cleaning the surfaces to remove the entirety of the polymer brush coating. This can be done through physical or chemical cleaning processes depending on the nature of the surface and the polymers involved. After cleaning, a second initiator can be deposited and used once again to grow brushes.^41^ Although this process maximizes the utility of the surface, exploring alternative strategies could eliminate the need for repetitive initiator deposition and surface cleaning for brush regrowth.

Aside from hindering degrafting reactions, the functionality of some macroinitiators can be preserved and used for further brush regrowth. Recent works have proven the reusability of PGMA-based macroinitiators by the repeated growth of polyester brushes from previously degraded samples.^18^ By using these brushes, surface modifications with a well-defined degradation that can be regrown on the same surface were enabled, moving toward a more circular approach to polymer brush growth.

Together with surface reusability, brush depolymerization is crucial for the development of truly recyclable brush coatings. This would involve the chemical recycling of the brush chains down to their monomer units, allowing their reuse to create more brush coatings. The chemical recycling of polymer brush coatings is a promising area of research that aligns with global efforts toward sustainability and waste reduction. However, it is also a field that requires further research, particularly in addressing technical challenges, which will be discussed in the following section.

## Moving Forward

The successful recycling of polymer brush coatings will have significant implications in various industries, particularly where they will be used extensively, such as in biomedical devices, sensors, and antifouling coating platforms.

While the core principles and some methodologies from the chemical recycling of bulk polymers can potentially be applied to polymer brushes, the distinct structural and functional properties of brushes require careful consideration and potentially novel approaches. This could involve unique catalysts, solvents, or reaction conditions to effectively break down the polymer chains without losing functional groups or contaminating the resulting monomers. Further research and development in this field is indispensable to address these challenges and harness the full potential of recycling these advanced materials.

Currently used techniques in the chemical recycling of bulk polymers, such as pyrolysis, hydrolysis, or enzymatic degradation,^25^ could potentially be adapted for polymer brushes. Only in the past few years, reports on the depolymerization by polymethacrylates in solution^42^ and solvent-free^43^ have been presented. Using relatively high temperatures (180–230 °C) and rapid depolymerization times (5–20 min), it was possible to recover high monomer fractions (60–99%) via both strategies. By applying this knowledge to grafted brushes, the reuse of degradation products derived from brushes may be possible. However, the effectiveness of these methods depends on the specific chemical structure and stability of the brush coating. For instance, the grafting density, thickness, and cross-linking of the polymer chains in brush coatings might affect the efficiency of these recycling processes.

Analytical techniques used in understanding the breakdown products of bulk polymers can be valuable in studying the degradation pathways of polymer brushes. This can aid in optimizing the recycling process and ensuring the purity of the recovered materials. Spectroscopic, X-ray, and chromatographic techniques used in the analysis of depolymerization of bulk polymers^42,43^ can be adjusted for brush coatings, as they are common techniques which have been successfully used on brush systems for other purposes. Other employed techniques in depolymerization analysis, such as thermal analysis or microscopy techniques, may require adjustments for their implementation on brush coatings.

In conclusion, although the implementation of circular polymer brush coatings presents unique economic and environmental challenges, it also offers the opportunity for sustainable material management. Balancing the potentially high complexity and costs with innovative methods and environmental benefits is the key to realizing the potential of this circular approach.
